# Impact of immunization against OxLDL on the pulmonary response to cigarette smoke exposure in mice

**DOI:** 10.1186/s12931-018-0833-9

**Published:** 2018-07-03

**Authors:** Maude Talbot, Mélanie Hamel-Auger, Marie-Josée Beaulieu, Morgan Gazzola, Ariane Lechasseur, Sophie Aubin, Marie-Ève Paré, David Marsolais, Ynuk Bossé, Mathieu C. Morissette

**Affiliations:** 1 Faculty of Medicine, Université Laval, Quebec City, Canada; 2 Quebec Heart and Lung Institute - Université Laval, 2725 Chemin Sainte-Foy, Quebec City, G1V 4G5 Canada; 3 Department of Medicine, Université Laval, 2725 Chemin Sainte-Foy, Quebec City, G1V 4G5 Canada

## Abstract

**Background:**

Cigarette smoke exposure can affect pulmonary lipid homeostasis and cause a progressive increase in pulmonary antibodies against oxidized low-density lipoproteins (OxLDL). Similarly, increased anti-OxLDL antibodies are observed in atherosclerosis, a pathology also tightly associated with smoking and lipid homeostasis disruption. Several immunization strategies against oxidized lipid species to help with their clearance have been shown to reduce the formation of atherosclerotic lesions. Since oxidized lipids are generated during cigarette smoke exposure, we investigated the impact of a prophylactic immunization protocol against OxLDL on the pulmonary effects of cigarette smoke exposure in mice.

**Methods:**

Mice were immunized systemically with a mixture of human OxLDL (antigen source) and AddaVax (adjuvant) or PBS alone prior to the initiation of acute (2 week) or sub-chronic (8 weeks) cigarette smoke exposure protocols. Anti-OxLDL antibodies were measured in the bronchoalveolar lavage (BAL) fluid and serum by direct ELISA. Pulmonary impacts of cigarette smoke exposure and OxLDL immunization were assessed by measuring BAL inflammatory cells, lung functions, and changes in lung structure and gene levels of matrix/matrix-related genes.

**Results:**

Immunization to OxLDL led to a marked increase in circulating and pulmonary antibodies against OxLDL that persisted during cigarette smoke exposure. OxLDL immunization did not exacerbate or reduce the inflammatory response following acute or sub-chronic exposure to cigarette smoke. OxLDL immunization alone had effects similar to cigarette smoke exposure on lung functions but OxLDL immunization and cigarette smoke exposure had no additive effects on these parameters. No obvious changes in lung histology, airspace or levels of matrix and matrix-related genes were caused by OxLDL immunization compared to vehicle treatment.

**Conclusions:**

Overall, this study shows for the first time that a prophylactic immunization protocol against OxLDL can potentially have detrimental effects lung functions, without having additive effects over cigarette smoke exposure. This work sheds light on a complex dynamic between anti-OxLDL antibodies and the pulmonary response to cigarette smoke exposure.

## Introduction

Tobacco smoking is well known to trigger a rapid and robust inflammatory response in the lungs along with progressive structural alterations. Mechanistically, cigarette smoking has a significant impact on pulmonary lipid homeostasis [1–4]. In fact, cigarette smoke rapidly causes lipid accumulation in pulmonary macrophages, a phenotype that persists following smoking cessation [3]. Interestingly, the interleukin (IL)-1α-dependent neutrophilia triggered by cigarette smoke exposure can be mimicked by the local delivery of oxidized lipids (oxidized low-density lipoprotein; OxLDL), suggesting that cigarette smoke-mediated generation of bioactive lipids could trigger what eventually becomes chronic inflammation [3].

In addition to the inflammatory response and structural damage, the pulmonary response to cigarette smoke exposure causes an increase in pulmonary antibodies with affinity for OxLDL [5]. Such antibodies can affect the pulmonary response to cigarette smoke, notably by promoting lipid and smoke particle uptake by pulmonary macrophages [5]. The roles of natural anti-OxLDL antibodies and B cells have been more extensively investigated in the pathogenesis of atherosclerosis. Depending on the approach and/or the B cell subset investigated, B cells and anti-OxLDL antibodies appear to have both pro- and anti-atherosclerotic effects [6].

While the biological role of natural anti-OxLDL antibodies on atherosclerosis progression remains controversial, therapeutic strategies using adjuvant-mediated immunization to increase the levels of anti-OxLDL antibodies have been explored. Several groups showed a beneficial impact of this type of approach to limit atherosclerotic processes in animals [7–10]. Since both the cigarette smoke-exposed lung and atherosclerotic lesions display local lipid homeostasis disruption, activated lipid-laden macrophages, progressive and chronic inflammation, gradual tissue alterations, spontaneous increase in anti-OxLDL antibodies, we hypothesized that increasing antibodies against OxLDL trough a vaccine-like process would affect the pulmonary response to cigarette smoke, at both immunological and functional levels. This would provide information on their biological relevance and possible a new therapeutic paradigm. Therefore, our main objective was to identify the impact of high anti-OxLDL antibodies, induced by adjuvant-mediated immunization against OxLDL, on the pulmonary immune and functional responses to cigarette smoke.

In this study, we successfully developed an immunization protocol that increased anti-OxLDL antibody levels in the lungs that remains effective during cigarette smoke exposure. The therapy did not exacerbate or reduce the inflammatory response to cigarette smoke in acute or sub-chronic exposures. OxLDL immunization alone had a significant impact on lung functions but cigarette smoke exposure had no additive effect in immunized animals. Altogether, this study shows for the first time that an immunization therapy against OxLDL does not impact the immune response to cigarette smoke exposure and suggest that it could have detrimental effects on pulmonary functions.

## Methods

### Mice

Six to eight weeks old female BALB/c mice were used in this study (Charles River, St-Constant, QC, Canada). Mice were housed according to the Canadian Council for Animal Care (CCAC) guidelines and Université Laval’s Animal Research Ethics Board approved all procedures (Animal utilization protocol #2014121–2).

### Immunization to OxLDL

The immunization cocktail consisted of a 1:1 mixture of CuSO_4_-oxidized low-density lipoprotein (OxLDL; 100 μg in 100 μL) from human plasma (BT-910X; Alfa Aesar, Ward Hill, MA, USA) as the antigen source and AddaVax™ (InvivoGen, San Diego, CA, USA) as the adjuvant. Mixture was made fresh before injection (mixed by up-and-down and tube inversion). Mice were injected intraperitonealy with 200 μL of the immunization cocktail three times at two-week intervals.

### Cigarette smoke exposure

Mice were exposed to the mainstream smoke of 3R4F cigarettes (University of Kentucky, Lexington, KY, USA) for 2 h a day, 5 days a week for 2 (acute) or 8 weeks (sub-chronic) using a well-characterized whole-body exposure system (SIU24 system, Promech Lab AB, Vintrie, Sweden). Control groups were exposed to room air.

### Pulmonary function assessment

Lung functions were assessed using the FlexiVent® (SCIREQ, Montreal, PQ, Canada). Mice were anesthetized with 100 mg/kg ketamine and 10 mg/kg xylazine; tracheostomized with an 18-gauge blunted needle, mechanically ventilated at a respiratory rate of 150 breaths/minutes and a tidal volume of 10 ml/kg, with a pressure limit of 30 cmH_2_O. Muscle paralysis was achieved using pancuronium (2 mg/kg, Sandoz, Boucherville, PQ, Canada) to prevent respiratory efforts during the measurement. The following sequence of measures was repeated three times: Deep inflation, Snapshot-150, Quick Prime-3 and Pressure/Volume-loop to obtain lung inspiratory capacity, compliance, resistance, Newtonian resistance, tissue damping, tissue elastance and the P-V loop.

### Sample collection and processing

When Flexivent was not performed, mice were anesthetized with isoflurane and euthanized by exsanguination. Lungs were removed from the chest cavity and the trachea canulated. The right lung was tied using suture strings, snap frozen in liquid nitrogen, and kept at − 80 °C. Bronchoalveolar lavage (BAL) was performed on the left lung by lavaging the lungs twice with 250 μl of PBS. Following BAL, the left lung was inflated with 10% formalin and embedded in paraffin for histological assessment. BAL total cell concentration was determined using a hemocytometer. BAL cells were pelleted at 800 x g, the cell-free BAL fluid (BALF) was collected and stored at − 80 °C. The cell pellet was resuspended in PBS to perform cytospins. Cytospins were stained (Diff-Quik; Fisher Scientific, Ottawa, ON, Canada) and differential counts performed using the Image J software by counting at least 300 cells per cytospin. Blood was collected, left to coagulate at 37 °C, and the serum separated by centrifugation and stored at − 80 °C.

### Measurement of OxLDL-specific antibodies

Wells of a 96-well plate were coated overnight with 2 μg/100 μL of human OxLDL (AlfaAesar) in PBS. Wells were blocked with 200 μL of PBS-0.05% Tween + 1% BSA (Sigma-Aldrich, Oakville, ON, Canada) for 1 h. Wells were incubated at room temperature with diluted serum or BALF (1:3 × 10^6^ and 1:10 [5], respectively, in PBS-0.05% Tween + 1% BSA) for 2 h. Wells were then washed 5 times with PBS-0.05% Tween. For detection, wells were incubated with the goat anti-mouse IgG + IgM + IgA H&L coupled to biotin (0.25 μg/mL in PBS-0.05% Tween + 1% BSA; AbCam, Toronto, ON, Canada) for 1 h, washed, incubated with Streptavidin-HRP (1:40; R&D systems, Minneapolis, MN, USA) for 30 min, washed and incubated with TMB substrate reagent BD OptEIA™ (BD Biosciences, San Jose, CA, USA) for 20 min. The reaction was stopped after 30 min with 2 N H_2_SO_4_ and the absorbance at 450 nm was read using Synergy H1 Hybrid Reader (BioTek, Winooski, VT, USA).

### Elisa

BAL fluid Monocyte chemoattractant protein-1 (MCP-1) concentrations were assessed using the mouse CCL2/JE/MCP-1 DuoSet® ELISA kit (R&D systems, Minneapolis, MN, USA) according to manufacturer’s instructions.

### Quantitative PCR

Lung lobes were homogenized in 1 ml of Trizol (Fisher Scientific) using PowerGen 125 polytron (Fisher Scientific). RNA was extracted according to manufacturer’s instructions. RNA concentration and purity was determined using Take3 Trio Micro-Volume plate and Synergy H1 Hybrid Reader (BioTek). RNA integrity was verified using agarose gel electrophoresis. Reverse transcription was performed on 1 μg of RNA using the iScript™ Advanced cDNA Synthesis Kit for quantitative PCR (qPCR) (Bio-Rad, Mississauga, ON, Canada) according to manufacturer instructions. *Cxcl5*, *spp1*, *ctgf*, *acta2*, *col1a1*, and *col3a1* lung mRNA expression was assessed by qPCR and normalized to *hprt* and *rplp0* reporter genes using the ∆∆Cq method. All reactions were performed in duplicate or triplicate using SsoAdvanced™ Universal SYBR® Green Supermix (Bio-Rad) and primers at 10 μM. The primers and qPCR conditions used for amplifications were optimised for each gene (*cxcl5* [NM_009141]: Fwr TTG TGT TGC TGT TCA CGC T - Rev. ATC ACC TCC AAA TTA GCG ATC A - annealing T° 59 °C; *spp1* [NM_001204203, NM_001204201, NM_009263, NM_001204202 and NM_001204233]: Fwr TCG TCA TCA TCG TCG TCC A – Rev. AGA ATG CTG TGT CCT CTG AAG - annealing T° 57°C; *ctgf* [ NM_010217]: Fwr TTG ACA GGC TTG GCG ATT – Rev. GTT ACC AAT GAC AAT ACC TTC T - annealing T° 60°C; *acta2* [ NM_007392]: Fwr CTG TTA TAG GTG GTT TCG TGG A – Rev. GAG CTA CGA ACT GCC TGA C - annealing T° 60°C; *col1a1* [ NM_007742]: Fwr CAT TGT GTA TGC AGC TGA CTT C – Rev. CGC AAA GAG TCT ACA TGT CTA G - annealing T° 60°C, and *col3a1* [ NM_009930] Fwr TCT CTA GAC TCA TAG GAC TGA C– Rev. TTC TTC TCA CCC TTC TTC ATC C- annealing T° 60°C, *hprt* [NM_013556]: Fwr AGC AGG TCA GCA AAG AAC T - Rev. CCT CAT GGA CTG ATT ATG GAC A - annealing T° 57 °C; *rplp0* [NM_007475.5]: Fwr ATC ACA GAG CAG GCC CTG CA - Rev. CAC CGA GGC AAC AGT TGG GT - annealing T° 57 °C). qPCR were performed using a Rotor-Gene 6000 series (QIAGEN, Toronto, ON, Canada) and data acquired/analysed with the on-board Rotor-Gene series Software version 1.7. All qPCR efficiencies were between 90 and 110%, with R^2^ values ranging between 0.97–1.00. The thermo-protocol was as follows: 95 °C for 3 min, followed by 40 cycles of 95 °C for 10 s and 57–60 °C for 30 s and followed by a melting curve.

### Lung histology and 2D airspace and tissue assessments

Paraffin-embedded lungs were cut at a thickness of 5 μm, and stained with hematoxylin and eosin (H&E). Pictures were taken using bright field microscopy with the Eclipse 6000 microscope (Nikon), Retiga Exi Aqua camera, and Qcapture pro 7 software (QImaging). For 2D morphological assessments, H&E-stained lung sections were scanned using the Hamamastu NanoZoommer 2.0 HT (C9600–12) (Hamamatsu Photonics K.K.). CT-Analyser software (Bruker) was used for the segmentation of the foreground from background to obtain binarised images for each individual lung section (3 sections/mouse lung). Integrated 2D analysis inside a region of interest (ROI) corresponding to the boundary of the binarised lung section images was made to obtain the percentage of airspace and tissue.

### Statistical analysis

Statistical differences were assessed using one-way ANOVA (> 2 groups) followed by post hoc Tukey’s multiple comparison tests. Two-sided unpaired Student’s t-tests were also used for 2-group comparisons and for complementary statistical assessments. Tests were performed using GraphPad Prism 7.

## Results

### Impact of recall numbers and cigarette smoke exposure on circulating and pulmonary anti-OxLDL antibody levels

**Fig. 1:**
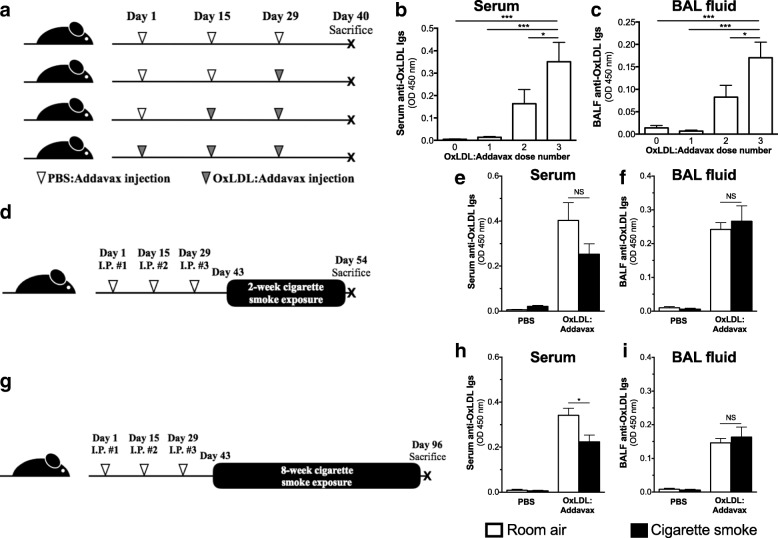
Impact of OxLDL immunization on circulating and pulmonary levels of anti-OxLDL antibodies and its interaction with cigarette smoke exposure. Six to eight weeks old female BALB/c mice (*n* = 5/group) were subjected to (**a**) PBS:Addavax or OxLDL:Addavax immunization on day 1, 15 and 29, and euthanized on day 40. Anti-OxLDL antibodies were measured by direct ELISA in (**b**) serum (dilution 1:3 × 10^6^) and (**c**) bronchoalveolar lavage fluid (BALF) (dilution 1:10 [5]). **d** Mice (*n* = 5/group) were injected with PBS alone or immunized against OxLDL (OxLDL:Addavax) and exposed to room air or cigarette smoke for 2 weeks. Anti-OxLDL antibodies were measured by direct ELISA in (**e**) the serum and (**f**) BALF. **g** Mice (n = 5/group) were injected with PBS alone or immunized against OxLDL (OxLDL:Addavax) and exposed to room air or cigarette smoke for 8 weeks. Anti-OxLDL antibodies were measured by direct ELISA in (**h**) the serum and (**i**) BALF. One-way ANOVA followed by Tukey’s multiple comparison tests *p < 0.05; ***p < 0.001; NS = not significantly different after two-sided Student T test

The immunization strategy using OxLDL and AddaVax™ caused a marked elevation of anti-OxLDL antibodies in both the circulation and the lungs. This increase in circulating and pulmonary anti-OxLDL antibodies was proportional to the number of injections (Fig. 1a-c). Since three injections led to higher antibody levels, this protocol was used to immunize mice prior acute (Fig. 1d) and sub-chronic (Fig. 1g) cigarette smoke exposure. Acute cigarette smoke exposure had no significant effects on serum or BAL fluid anti-OxLDL antibody levels (Fig. 1e and f). However, sub-chronic exposure to cigarette smoke significantly reduced serum anti-OxLDL antibody levels (Fig. 1h) while having no effects on BAL fluid levels (Fig. 1i). Altogether, these data show that the OxLDL immunization strategy markedly increases anti-OxLDL antibodies in the lungs and circulation; sub-chronic cigarette smoke exposure only affecting circulating levels.

### Impact of OxLDL immunization on the pulmonary inflammatory response to cigarette smoke exposure

**Fig. 2:**
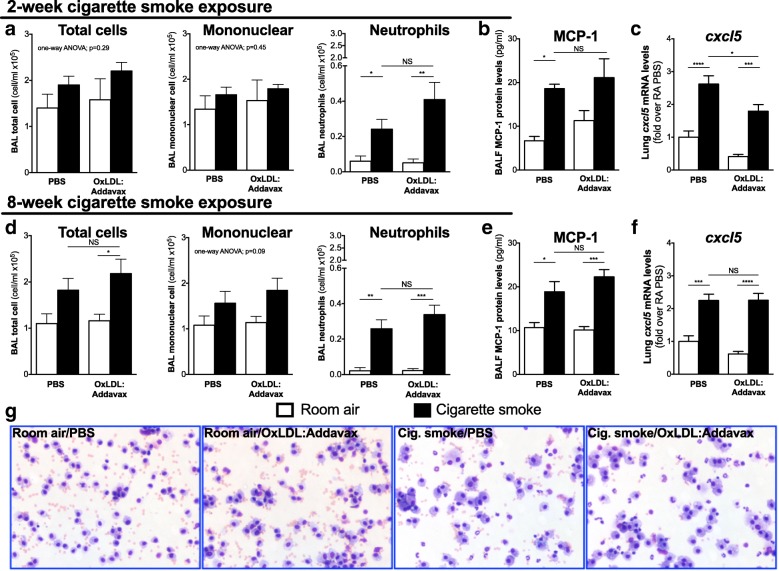
OxLDL immunization does not exacerbate the pulmonary inflammatory response to cigarette smoke exposure. Six to eight weeks old female BALB/c mice (*n* = 5/group) were injected with PBS alone or immunized against OxLDL (OxLDL:Addavax) and exposed to room air or cigarette smoke for 2 (**a-c**) or 8 weeks (**d-f**). Bronchoalveolar lavage (BAL) total cell numbers and differential cell (mononuclear cells and neutrophils) counts were assessed in (**a**) the 2-week and (**d**) the 8-week exposure protocols. BAL fluid MCP-1 protein levels and lung *cxcl5* mRNA levels were also measured in (**b-c**) the 2-week and (**e-f**) the 8-week exposure protocols. **g** Representative cytospins from total BAL cells stained with haematoxylin and eosin. One-way ANOVA followed by Tukey’s multiple comparison tests *p < 0.05; **p < 0.01; ***p < 0.001; ****p < 0.0001; NS = not significantly different after two-sided Student T test

To investigate how immunization to OxLDL could affect the pulmonary inflammatory response to cigarette smoke exposure, we assessed BAL total cell number and cell differentials. BAL MCP-1 and *cxcl5* lung mRNA levels were assessed as they are both rapidly induced by cigarette smoke exposure and linked to monocyte/macrophage and neutrophil recruitment [5, 11]. As expected, both acute and sub-chronic exposure to cigarette smoke led to a marked increased in BAL neutrophils (Fig. 2a and d), MCP-1 levels (Fig. 2b and e), and *cxcl5* lung mRNA (Fig. 2c and f). Immunization to OxLDL had no significant effect on smoking-induced neutrophilia (Fig. 2a and d) or MCP-1 levels (Fig. 2b and e). In the acute protocol, mRNA levels of *cxcl5* were significantly lower in immunized animals exposed to cigarette smoke (Fig. 2c), but did not remain altered following a longer exposure protocol (Fig. 2f). Finally, no significant changes were observed when looking at the morphology of pulmonary macrophages (Fig. 2g). These data show that the inflammatory response triggered by an acute or sub-chronic exposure to cigarette smoke is not significantly affected by anterior immunization to OxLDL.

### Impact of OxLDL immunization on smoking-induced changes in lung functions

**Fig. 3:**
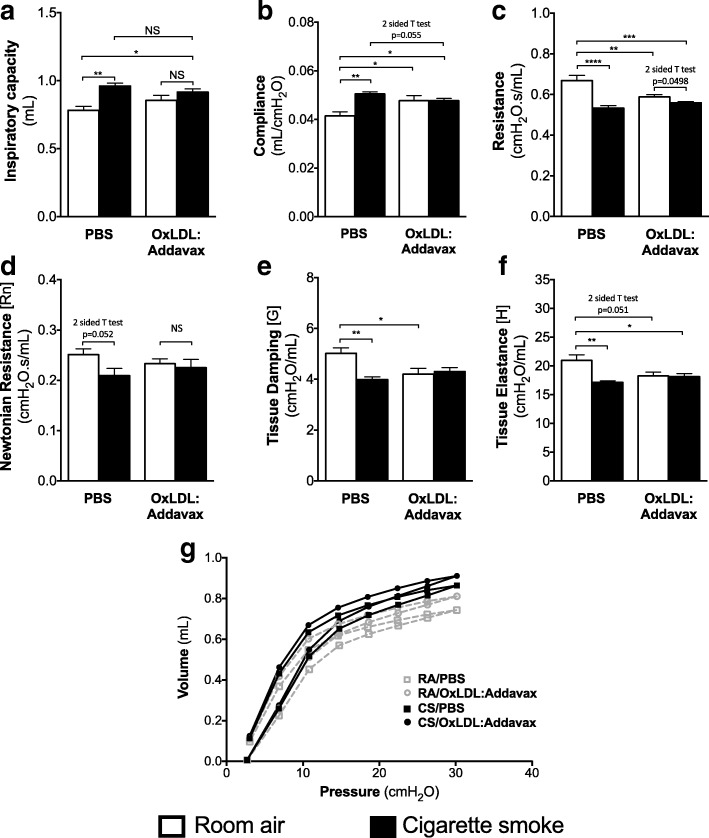
Impact of OxLDL immunization on cigarette smoke-induced alterations in lung functions. Six to eight weeks old female BALB/c mice (*n* = 5/group) were injected with PBS alone or immunized against OxLDL (OxLDL:Addavax) and exposed to room air or cigarette smoke for 8 weeks. Pulmonary mechanics were assessed using the FlexiVent showing (**a**) inspiratory capacity (from deep inflation), **b** compliance (from Snap Shot 150), **c** resistance (from Snap Shot 150), **d** Newtonian resistance (from Quick Prime 3), **e** tissue damping (from Quick Prime 3), **f** tissue elastance (from Quick Prime 3) and **g** pressure-volume (P-V) loop. One-way ANOVA followed by Tukey’s multiple comparison tests *p < 0.05; **p < 0.01; ***p < 0.001; ****p < 0.0001; NS = not significantly different after two-sided Student T test

To assess the impact of OxLDL immunization on smoking-induced changes in lung functions, we performed FlexiVent® analysis in animals subjected to the 8-week sub-chronic cigarette smoke exposure protocol. As expected, cigarette smoke affected the lung parenchyma mechanical properties of PBS-treated mice, increasing inspiratory capacity and compliance, and reducing resistance, Newtonian resistance, tissue damping and tissue elastance (Fig. 3). On its own, OxLDL immunization caused lung function changes in room air-exposed mice very similar to those caused by cigarette smoke exposure (Fig. 3), increasing compliance, and reducing resistance, tissue damping and tissue elastance, suggesting the immunization itself can impact lung parenchyma mechanics. Interestingly, cigarette smoke exposure did not exacerbate the changes in lung functions caused by OxLDL immunization (Fig. 3), leaving the room air and cigarette smoke-exposed mice immunized against OxLDL with similar lung functions. This data shows that OxLDL immunization alone can affect lung functions, although cigarette smoke exposure and OxLDL immunization do not have additive effects.

### Impact of OxLDL immunization on pulmonary structure

**Fig. 4:**
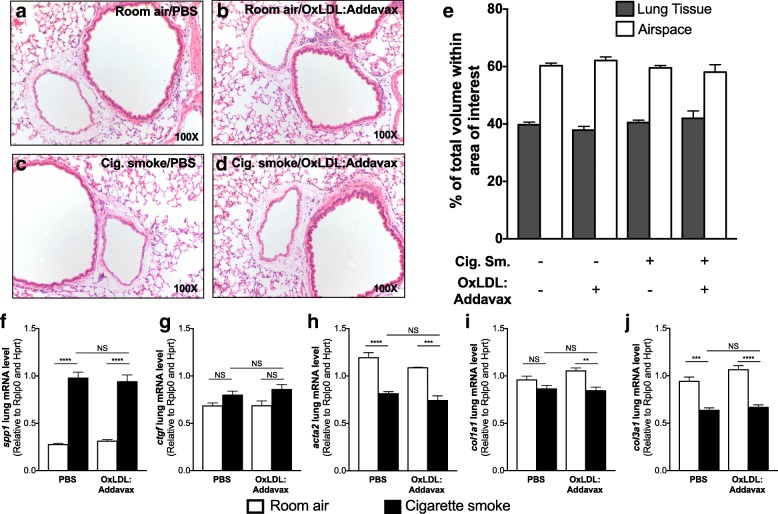
OxLDL immunization does not impact cigarette smoke-induced histological changes. Six to eight weeks old female BALB/c mice (n = 5/group) were injected with PBS alone or immunized against OxLDL (OxLDL:Addavax) and exposed to room air or cigarette smoke. Representative lung histological pictures of mice treated with PBS alone or immunized against OxLDL (OxLDL:Addavax) and exposed to room air or cigarette smoke for 8 weeks. **a-d** Representative fields from lung sections stained with haematoxylin & eosin. **e** Airspace and lung tissue area were determined from scanned lung sections (3 sections/mouse lung). Whole lung (**f**) osteopontin (*spp1*), **g** connective tissue growth factor (*ctgf*), **h** alpha 2 smooth muscle actin (*acta2*), **i** collagen type 1 alpha 1 chain (*col1a1*), and **j** collagen type 1 alpha 3 chain (*col1a3*) mRNA levels was assessed by qPCR. One-way ANOVA followed by Tukey’s multiple comparison tests **p < 0.01; ***p < 0.001; ****p < 0.0001; NS = not significantly different after two-sided Student T test

With OxLDL immunization having a significant impact on lung functions, we investigated the structure of the lungs with respect to bronchial epithelium, vessels, and parenchyma. We also performed qPCR to investigate mRNA levels of matrix and matrix-related genes, which could explain why lung functions are altered by OxLDL immunization. With regard to lung structure, we did not observe any major change caused by immunization to OxLDL in room air or cigarette smoke-exposed mice (Fig. 4a-d). Quantification of airspace and lung tissue area showed no loss in pulmonary tissue caused by the immunization (Fig. 4e). Cigarette smoke significantly affected mRNA levels of *spp1* (osteopontin), *col1a1* (Collagen Type I Alpha 1 Chain), *col3a1* (Collagen Type III Alpha 1 Chain), and *acta2* (alpha 2 smooth muscle actin), but not *ctgf* (connective tissue growth factor) (Fig. 4f-j). However, no specific effects of OxLDL immunization were observed.

## Discussion

We have previously shown that cigarette smoke exposure elicits a local increase in anti-OxLDL antibodies in the lungs and that a monoclonal antibody with affinity for oxidized lipids does not appear to exacerbate the inflammatory response while promoting lipid and particle uptake by pulmonary macrophages [5]. In this study, we developed an immunization protocol against OxLDL that leads to high anti-OxLDL antibody levels in the lungs. Our results unravel a complex interaction between anti-OxLDL antibodies, cigarette smoke exposure and their impact on the pulmonary response and lung function alterations.

The systemic immunization protocol was developed in mice using AddaVax as adjuvant and human OxLDL as the antigen source and lead to a marked increase in anti-OxLDL antibodies in both the circulation and the lungs. Similar immunization strategies have been used in animal models of atherosclerosis [7–10] and, to the best of our knowledge, we are the first to show that such a treatment also leads to an increase in anti-OxLDL antibodies in the lungs. Cigarette smoke exposure, most notably the 8-week sub-chronic protocol, led to a reduction in circulating anti-OxLDL antibodies while pulmonary levels remained unaffected. In the lungs, cigarette smoke leads to an increase in immunoglobulins [12]. Thus, it is possible that this overall increase in pulmonary immunoglobulins compensates for the reduction in the circulation, leading to similar pulmonary levels of anti-OxLDL antibodies between room air and cigarette smoke-exposed mice.

To investigate the impact of the OxLDL immunization protocol on cigarette smoke-induced lung inflammation, we used a well-characterized whole-body exposure system. Overall, the anti-OxLDL immunization protocol did not appear to have any marked effects on the inflammatory response. Previous work with an anti-oxidized phosphatidylcholine antibody (clone E06) also showed little effects on the cellular inflammatory response to cigarette smoke exposure [5]. Oxidized phospholipid species are well known for their ability to trigger inflammatory processes [13]. Moreover, intranasal administration of OxLDL has been shown to cause lipid accumulation in pulmonary macrophages as well as an IL-1α-dependent neutrophilia similar to what is observed during cigarette smoke exposure [3]. While it remains unclear why the OxLDL immunization does not impact the acute or sub-chronic inflammatory response to cigarette smoke, our data clearly show that an immunization therapy using OxLDL as an antigen source does not exacerbate the immune response to cigarette smoke.

Cigarette smoking is well known to impact lung functions over time. This phenomenon is also observed in pre-clinical models where cigarette smoke exposure leads to an increase in lung volume and compliance as well as a reduction in pulmonary resistance and elastance. To investigate the impact of OxLDL immunization on lung function alterations caused by cigarette smoke exposure, analyses were performed at the end of the 8-week cigarette smoke exposure protocol. As expected, cigarette smoke exposure increased lung volume and compliance, and reduced pulmonary resistance and elastance. Interestingly, lung functions of animals exposed to room air and immunized against OxLDL were very similar to those of cigarette smoke-exposed animals. While the aforementioned observation is relatively surprising, we also observed that OxLDL immunization and cigarette smoke exposure had no additive effects. This suggests that OxLDL immunization and cigarette smoke exposure trigger redundant mechanisms leading to lung function alterations.

To identify clues that could explain functional changes caused by OxLDL immunization, we investigated its impact on the lung microscopic structure as well as on the expression of key genes coding for matrix and matrix-related proteins. We could not observe any marked differences between the cigarette smoke-exposed mice that received the therapy or the vehicle, suggesting that the immunization therapy does not affect visible histological alterations or changes in matrix and matrix-related gene expression. Emphysema-like changes could not be observed at this stage, as a chronic exposure of 4 to 6 months is usually required to observe such changes. Further investigations are required to fully decipher the impact of OxLDL immunization on the pulmonary structure and physiology.

While we do not know the mechanisms by which immunization against OxLDL alters lung functions, we can speculate on potential candidates. Since OxLDL immunization does not induce any kind of pulmonary immune response, we can confidently say that the changes in lung functions are not due to an inflammatory response. Since OxLDL contains numerous species of oxidized phospholipids, increased anti-OxLDL antibodies could interact with the pulmonary surfactant, potentially changing its dynamic, which could possibly result in the observed reduction in resistance. The mechanisms behind this phenomenon, and the fact that it is not additive to cigarette smoke exposure, definitely remain puzzling.

This study has some limitations. The immunization protocol was performed prophylactically prior to cigarette smoke exposure. It is not guaranteed that similar observations would be made in a therapeutic setting when immunization is initiated after the initiation of cigarette smoke exposure. While immunization against OxLDL was performed, we do not currently know the specific nature of the antigens targeted and that are mediating the physiological effects. Such investigations would require considerable scientific efforts but would be of great interest.

## Conclusion

Overall, this study shows for the first time that a prophylactic immunization protocol against OxLDL can impact lung functions, although having no additive effect over cigarette smoke exposure. This pre-clinical study is worth pursuing and further investigations are required to refine the technology and improve our understanding of the cellular and molecular mechanisms at play.

## Abbreviations

*acta2*: Actin, Alpha 2, Smooth Muscle, Aorta
ANOVA: Analysis of variance
BAL: Bronchoalveolar lavage
BALF: Bronchoalveolar lavage fluid
BSA: Bovine serum albumin
*col1a1*: Collagen Type I Alpha 1 Chain
*col3a1*: Collagen Type 3 Alpha 1 Chain
*ctgf*: Connective tissue growth factor
ELISA: Enzyme-linked immunosorbent assay
H&E: Haematoxylin and Eosin
IL: Interleukin
MCP-1: Monocyte chemoattractant protein 1
OxLDL: Oxidized low density lipoprotein
PBS: Phosphate-buffered solution
qPCR: Quantitative polymerase chain reaction
*spp1*: Secreted Phosphoprotein 1
